# Cytotopic (Cyto-) IL-15 as a New Immunotherapy for Prostate Cancer: Recombinant Production in *Escherichia coli* and Purification

**DOI:** 10.3389/fmolb.2021.755764

**Published:** 2021-10-27

**Authors:** Ana M. Esteves, Efthymia Papaevangelou, Dorota Smolarek, Prokar Dasgupta, Christine Galustian

**Affiliations:** ^1^ Peter Gorer Department of Immunobiology, School of Immunology and Microbial Sciences, King’s College London, Guy’s Hospital, London, United Kingdom; ^2^ Urology Centre, Guy’s Hospital, London, United Kingdom

**Keywords:** IL-15, *Escherichia coli*, protein purification, cytotopic modification, prostate cancer, immunotherapy

## Abstract

Interleukin-15 (IL-15) is a cytokine previously suggested as a potential immunotherapy for cancer treatment. IL-15 can effectively reduce tumor growth in many preclinical tumor models including prostate cancer. This is due to its ability to expand and activate immune cells, such as CD8^+^ T cells and natural killer cells. To increase the potency of IL-15, we have engineered a protein variant that can be modified to localize and be retained in tissues where it is administered. However, the production of recombinant IL-15, the purity, and correct refolding of the final protein is not always ideal. In the current study, we aimed to optimize the methodology for production and purification of a modified recombinant human IL-15 and investigate the efficacy of the produced protein in the treatment of prostate tumors. Human IL-15 with its polypeptide sequence modified at the C-terminus to enable thiol conjugation with membrane localizing peptides, was produced in *E. coli* and purified using mild denaturing conditions (2M urea) from a washing step or from solubilization of inclusion bodies. The purified protein from the wash fraction was conjugated to a myristoylated peptide to form a membrane-localizing IL-15 (cyto-IL-15). The efficacy of cyto-IL-15 was investigated in subcutaneous TRAMP-C2 prostate tumors in mice and compared with cyto-IL-15 derived from protein purified from inclusion bodies (cyto-IL-15 Gen). When mild denaturing conditions were used for purification, the largest amount of IL-15 was collected from the wash fraction and a smaller amount from inclusion bodies. The protein from the wash fraction was mainly present as a monomer, whereas the one from inclusion bodies formed homodimers and higher complexes. After cytotopic modification, the purified IL-showed great efficacy in delaying prostate tumor growth (∼50%) and increased mice survival by ∼1.8-fold compared with vehicle. This study demonstrates an alternative, inexpensive and efficient method to produce and purify a modified version of IL-15 using mild denaturing conditions. This IL-15, when cytotopically modified, showed great efficacy as a monotherapy in prostate tumors in mice further highlighting the potential of IL-15 as a cancer immunotherapy.

## Introduction

Interleukin-15 (IL-15), a protein with a molecular weight of 14–15 kDa, is a member of the four α-helix bundle family of cytokines that is involved in the proliferation and activation of natural killer (NK) and T cells (Waldmann, 2006). IL-15 mediates its functions through the IL-15Rα-chain, IL-2/IL-15Rβ-chain and the common cytokine receptor gamma chain (γ_c_). IL-15 is presented by IL-15Rα to the IL-2/IL-15Rβ/γ_c_ heterodimeric receptor on the surface of responding cells to initiate signaling (Waldmann et al., 2001).

During the past years, IL-15 has been considered one of the most promising cytokines in the treatment of cancer due to its ability to enhance the anti-tumoral response of CD8^+^ T and natural killer (NK) cells in pre-clinical studies (Klebanoff et al., 2004; Teague et al., 2006). Our laboratory has shown that IL-15 is the only protein among a panel of several cytokines that was able to expand and activate immune cells *in vitro*; this effect was increased when prostate cancer cells were present (Sakellariou et al., 2020). We have also shown that IL-15 was more potent in activating and expanding NK cells than IL-2 (Watkinson et al., 2020). Moreover, in *in vivo* studies, we have shown induction of cell death and improved mice survival when a membrane-localizing cytotopically modified IL-15 was injected directly into prostate tumors (Papaevangelou et al., 2020).

The interest demonstrated by the academia and industry in IL-15 makes it an attractive target for the recombinant production in large-scale in a cost-effective manner. Recombinant production of IL-15 has been attempted in several expression systems, such as mammalian cells (Jalah et al., 2007), yeast (Pettit et al., 1997; Hanick et al., 2007), and *E. coli* (Ward et al., 2009; Vyas et al., 2012). However, recombinant expression of IL-15 is not trivial as the protein tends to be produced as an insoluble protein, forming inclusion bodies (Ward et al., 2009; Vyas et al., 2012). Formation of inclusion bodies is a common problem in the field of protein production and it results from the incapacity of the protein to fold in its native-like structure (Ramon et al., 2014). Protein with the correct folding can be obtained from inclusion bodies, however this process is not ideal since only a small fraction of the protein typically refolds into its native-like structure. Protein is usually recovered from inclusion bodies using denaturing agents, which disrupt the secondary and/or tertiary structure of the proteins to later assume the correct structure by refolding. Strong denaturing agents usually disrupt both secondary and tertiary structures while mild denaturing agents disrupt only the tertiary structure (Singhvi et al., 2020).

In a previous study carried by our laboratory, the efficacy of a membrane-localizing cytotopically modified IL-15 to treat prostate tumors *in vivo* was examined (Papaevangelou et al., 2020). This study was challenging since pre-clinical assays demand higher quantities of the target drug and, only a small fraction (∼10%) of the purified recombinant IL-15 was suitable to be used *in vivo*. Therefore, in the current study, we aimed to improve the recombinant production and purification of IL-15 modified at the C-terminus of the polypeptide chain with a linker sequence (mod IL-15–Patent WO2021/058973), which was then further conjugated with a bis-myristoylated peptide *via* a disulfide bridge (cytotopic modification) to obtain a membrane localizing IL-15, without compromising the activity and effectiveness of the protein. IL-15 was initially expressed in *E. coli* and purified by affinity chromatography. Protein was then recovered from a washing step using mild denaturing conditions (mod IL-15) and then cytotopically modified to allow localization at cell membranes (cyto-IL-15). Protein was also recovered from inclusion bodies (designated as mod IL-15 Gen), and when cytotopically modified it was designated as cyto-IL-15 Gen. The binding efficiency and the activity of these proteins were investigated *in vitro*. Moreover, the efficacy of different doses of cyto-IL-15 was explored *in vivo* in a syngeneic subcutaneous prostate cancer mouse model.

## Materials and Methods

### Cloning and Expression of Modified IL-15

The nucleotide sequence encoding a modified version of human IL-15 (Papaevangelou et al., 2020), was cloned into the expression vector pET30a (Genscript Corporation, New Jersey, United States) using the restriction sites *Nde*I and *Hind*III. This modified version of human IL-15 consists of the full mature coding sequence of human IL-15 (amino acids 49–162), a peptide linker, six histidines to facilitate purification, and a cysteine at the C-terminus, producing a polypeptide with 17.4 kDa. The recombinant plasmid was transformed into chemically competent *E. coli* BL21 star (DE3) (ThermoFisher Scientific, Dartford, United Kingdom) by heat shock and a positive selection of transformed cells was carried out using Luria broth/agar plates supplemented with 30 μg/ml of kanamycin (Sigma-Aldrich, Dorset, United Kingdom). Positive clones were selected and cultivated at 37°C with agitation at 180 rpm in 2L Erlenmeyer flasks containing 440 ml of terrific broth (TB) medium supplemented with 30 μg/ml kanamycin. When the optical density (OD) at 600 nm reached 0.6, cells were cooled down for approximately 15 min at 4°C, treated with 0.5 mM isopropyl β-d-1-thiogalactopyranoside (IPTG) and allowed to grow further at 16°C for 16 h keeping agitation at 180 rpm. Cells were harvested by centrifugation at 2,500 × *g* for 10 min.

### Purification of Modified IL-15 From Inclusion Bodies Using Mild Versus Strong Solubilisation Conditions

**FIGURE 1:**
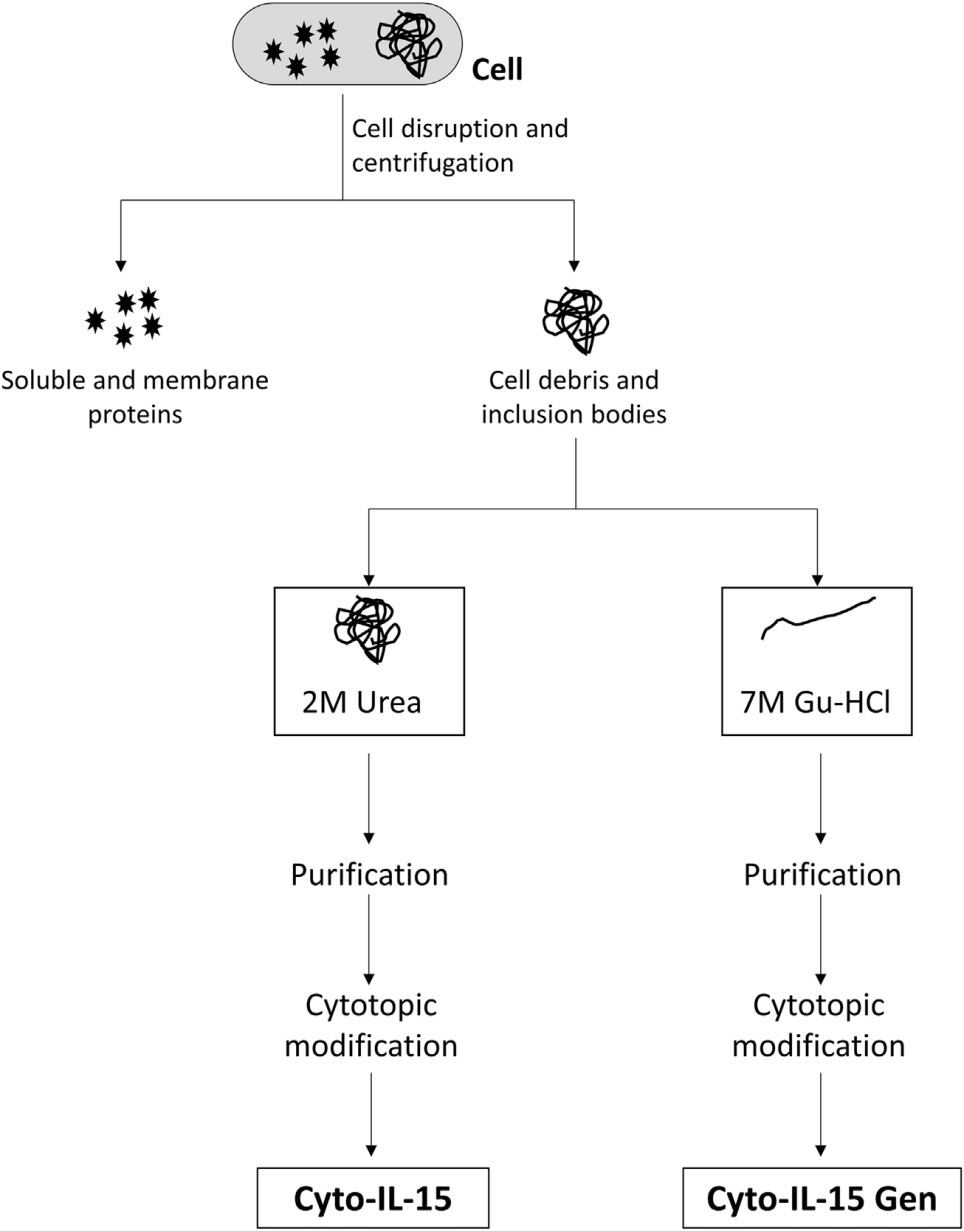
Schematic representation of the two solubilisation methods used in this study.

Cell pellets were resuspended in 100 mM Tris/HCl pH 8.0 containing 1 mM EDTA, 100 mM NaCl, 5% glycerol, 5 mM dithiothreitol (DTT), and protease inhibitor cocktail according to the manufacturer’s instructions (Sigma-Aldrich) and disrupted in a cell homogenizer EmulsiFlex-C5 (Avestin, Ottawa, ON, Canada). DTT and protease inhibitor were added to the buffer immediately before cell disruption. Cell debris were collected by centrifugation (12,000 × *g*, 45 min), the supernatant was discarded, and cell debris were used to purify modified IL-15 from inclusion bodies using two approaches: using mild denaturing conditions 1) and strong denaturant 2). For the first approach, debris were carefully resuspended in 100 mM Tris/HCl pH 8.0 containing 1 mM EDTA, 5% glycerol, 100 mM NaCl, 1 mM DTT, 2M urea, and 2% Triton X-114 (wash buffer, mild denaturing conditions) and centrifuged (12,000 × *g*, 30 min). Supernatant from the washing step was kept. Pellet was resuspended again with wash buffer and centrifuged as described above for a total of three washes. Supernatant from the three washes was immediately loaded into a Histrap HP column (GE Healthcare, Little Chalfont, United Kingdom) with the help of a chromatography ÄKTA Purifier system (GE Healthcare, Little Chalfont, United Kingdom), while the pellet was further used to solubilize IL-15 from inclusion bodies using a strong denaturant (second approach). Briefly, pellet was resuspended in 50 mM Tris/HCl pH 8.0 containing 300 mM NaCl, 7M Gu-HCl, and 1% Triton X-114 (solubilization buffer) and incubated overnight to solubilize remaining IL-15 in inclusion bodies. Solubilized IL-15 was applied into a Histrap HP column as well. Prior to loading of either of the samples into the Histrap, the column was equilibrated with 50 mM Tris pH 8.5, 8 M urea, and 1% Triton X-114. Modified IL-15 was eluted with 50 mM Tris pH 8.5, 8 M urea and a gradient of imidazole from 0 to 500 mM in 20 column volumes for 20 min at a flow rate of 1 ml/min. Fractions containing pure modified IL-15 were pooled together and dialyzed overnight against 50 mM Tris pH 8.5 containing 100 mM NaCl and 10% glycerol to allow refolding of the recombinant protein. Preparative size exclusion chromatography for further purification was attempted without success as most of the protein was lost during this process. Two batches of modified IL-15 were obtained at the end of the purification: modified IL-15 from the washings with mild denaturing buffer and modified IL-15 from solubilization of inclusion bodies (Figure 1).

### Cytotopic Peptide

**FIGURE 2:**
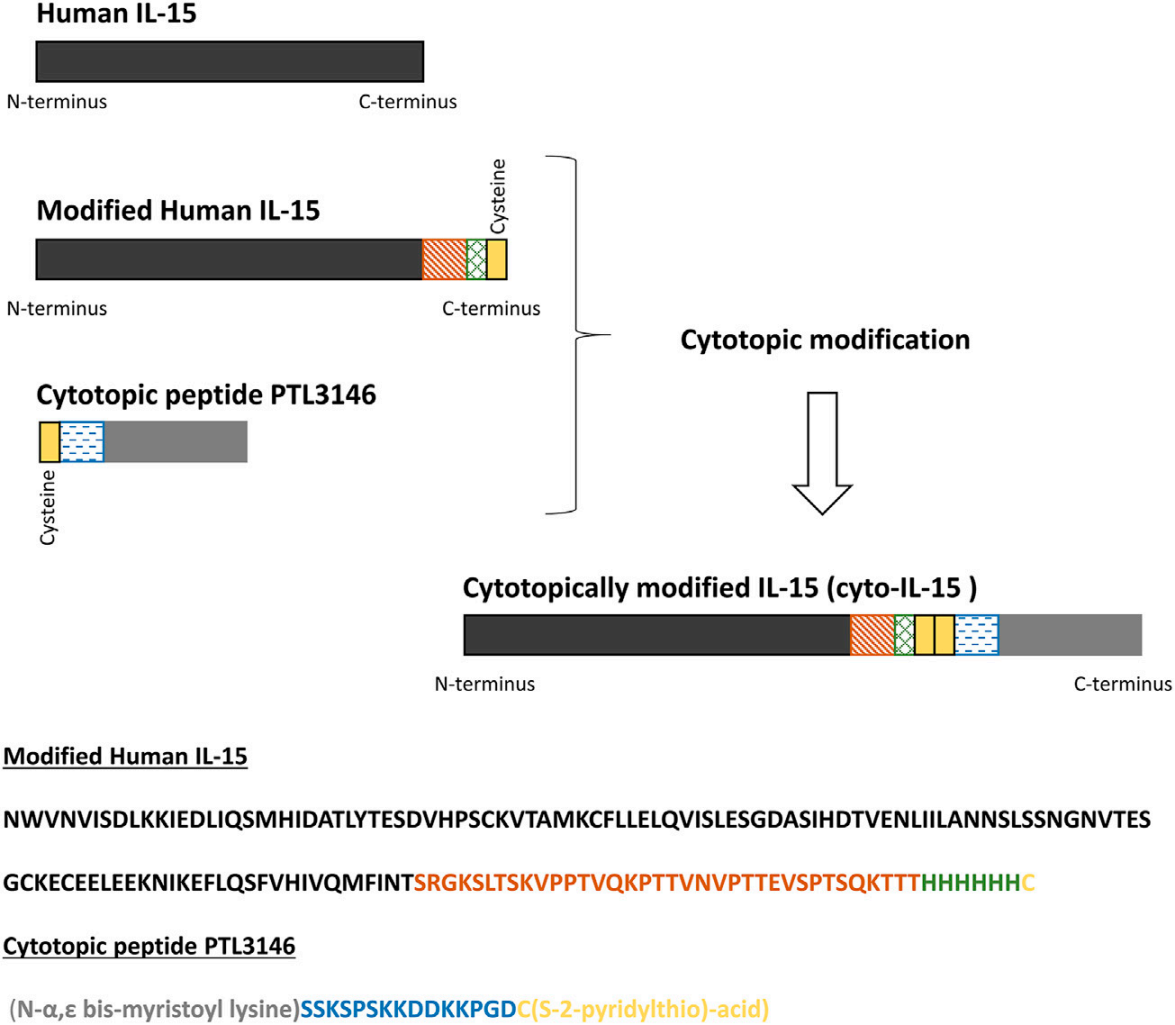
Schematic representation of modified human IL-15, the cytotopic peptide PTL3146, and the final cytotopically modified IL-15 (cyto-IL-15). In this work, a modified human IL-15 was expressed in *E. coli*. The recombinant protein consists of human IL-15 (dark grey), a linker (orange), a histidine tag to help with protein purification (blue), and a cysteine residue (yellow). The cytotopic peptide contains a cysteine residue as well (yellow), a positively charged peptide (blue) to interact with the cell membrane and two myristoyl groups (light grey).

The bis-myristoylated peptide (2.36 kDa) (named PTL3146) consists of two fatty acid myristoyl chains attached to the N-terminus of the L-lysine residue, the first amino acid of a positively charged peptide which is followed by an activated thiol group (Karegli et al., 2017). The sequence is provided in Figure 2. The thiol group forms a disulfide bridge with the free cysteine within the protein of interest while the myristoyl chains allow the protein of interest to insert spontaneously into the phospholipid bilayer upon interaction with cell surfaces. The positively charged peptide interacts with the negatively charged membrane.

### Cytotopic Modification of Purified IL-15

The purified IL-15 was linked at the C-terminus *via* the free cysteine residue with the cytotopic molecule PTL3146 (see Figure 2). The cytotopic modification of IL-15 was carried out as previously described (Papaevangelou et al., 2020) with a few changes: Recombinant modified IL-15 in 50 mM Tris/HCl pH 8.5 supplemented with 100 mM NaCl and 10% glycerol at a concentration of approximately 0.5 mg/ml was incubated with 50 µM tris(2-carboxyethyl)phosphine (TCEP) (Sigma-Aldrich) for 1 h at 4°C with gentle rotation. The excess of TCEP was removed by gel filtration (G25 column from GE Healthcare) and reduced IL-15 was immediately incubated with PTL3146 at a molar ratio of 1:1 protein to PTL3146 for 16 h at 4°C with gentle rotation. Unbound PTL3146 was removed by dialysis overnight against endotoxin-free phosphate buffered saline (PBS) pH 7.4 (Severn Biotech Ltd., Kidderminster, United Kingdom). The cytotopically modified IL-15 was termed cyto-IL-15 in the present study and a patent has been published for the modified IL-15 sequence (WO2021/058973). The presence of endotoxins in a sample of cyto-IL-15 was investigated using the Pierce Chromogenic Endotoxin Quant Kit (ThermoFisher). A value of 0.023 EU/μg of protein was obtained. Total protein concentration was estimated using a Pierce BCA protein assay Kit (ThermoFisher) according to manufacturer’s instructions, while concentration of IL-15 was measured using a human IL-15 ELISA MAX (435104, Biolegend, London, United Kingdom) according to manufacturer’s instructions. The same process was used to attach the modified IL-15 obtained from inclusion bodies (produced by Genscript Corporation) with the PTL3146 cytotopic peptide. In this study we refer to this batch as cyto-IL-15 Gen.

### SDS-PAGE Electrophoresis and Western Blot Analysis

Purity and quality of the protein was analysed by SDS-PAGE and western blot. Approximately 3 or 0.5 µg of total protein was loaded into a 4–12% NuPAGE Bis-Tris mini gel for either Coomassie staining or western blot analysis, respectively. For western blot analysis, samples were transferred to a nitrocellulose membrane using an iBlot transfer stack (ThermoFisher). Non-specific binding was prevented by blocking the membrane with 3% bovine serum albumin (Sigma-Aldrich). For the detection of IL-15, a commercially available mouse anti-human IL-15 antibody (1/20,000 dilution, PromoCell, Heidelberg, Germany) was used, while PTL3146 was identified using a rabbit polyclonal antibody produced by Eurogentec (Belgium, Brussels) (and purified *in house*)*,* that recognizes the positively charged peptide (Figure 2, amino acids labeled in blue) (1/3,000 dilution). Incubation with a goat anti-mouse horseradish peroxidise (HRP)-conjugated secondary antibody (1/1,000 dilution, Dako, Agilent Technologies, Stockport, United Kingdom) allowed the identification of either IL-15 or PTL3146 using an ECL Western Blotting Detection reagent (GE Healthcare).

### Cell Binding Assay

The ability of cytotopically modified IL-15 to bind to cell membranes *via* the PTL3146 peptide was investigated using murine red blood cells (RBCs) as these cells do not express cell surface receptors for IL-15. Fresh cells were diluted 1/400 in PBS pH 7.4 containing 2% fetal calf serum (wash buffer) and centrifuged at 300 × *g* for 5 min. The cell pellet was resuspended in residual buffer and incubated for 20 min with 1 µg of cytotopic IL-15 at room temperature. A control was performed where cytotopic IL-15 was replaced by modified IL-15 before cytotopic modification. Cells were washed with wash buffer and incubated for 30 min in the dark at 4°C with a mouse monoclonal anti-human IL-15 antibody conjugated with phycoerythrin (PE) (Bio-techne Ltd., Abington, United Kingdom). Cells were resuspended in PBS pH 7.4 containing 2% fetal calf serum and samples were analysed by flow cytometry using a FACSCalibur flow cytometer (BD Biosciences, Berkshire, United Kingdom). All data were analysed using FlowJo software (FlowJo LLC, Oregon, United States).

### Cell Culture

Murine cytotoxic T lymphocyte CTLL-2 cells, obtained from European Collection of Authenticated Cell Cultures, were maintained in RPMI-1640 medium with 2 mM L-glutamine, 1% antibiotic antimycotic solution, 0.2% gentamicin (all from Sigma-Aldrich) and 10% fetal bovine serum (ThermoFisher) (RPMI complete medium), supplemented with 10% T-STIM culture supplement with Concanavalin A (ThermoFisher). Transgenic adenocarcinoma of the mouse prostate (TRAMP)-C2 cells, obtained from American Type Culture Collection (ATCC, Teddington, United Kingdom), were maintained in Dulbecco’s Modified Eagle’s culture medium (DMEM) supplemented with 2 mM L-glutamine, 1% antibiotic antimycotic solution, 0.2% gentamicin, 100 U/ml penicillin, 0.2 mg/ml streptomycin, 5 μg/ml insulin, 0.01 nM dihydrotestosterone (all from Sigma-Aldrich), 5% fetal bovine serum (FBS) (ThermoFisher) and 5% NuSerum IV culture supplement (ThermoFisher). All cells were kept in a humidified atmosphere with 5% CO_2_ at 37°C and were negative for *mycoplasma* infection, which was tested frequently using LookOut *Mycoplasma* PCR (Sigma-Aldrich).

### Cell Proliferation Assay

The activity of IL-15 was investigated using a CTLL-2 cell proliferation assay. CTLL-2 cells were incubated for 4 h in phenol-free RPMI complete medium without T-STIM. Subsequently, 3.5 × 10^4^ cells/well were seeded in 96-well plates and treated with mod IL-15 or cytotopically-modified IL-15 at a range of 0–2.5 ng/ml concentration. Commercial IL-15, which is recombinant human IL-15 (without a C-terminus linker) was purchased from PeproTech EC Ltd. (London, United Kingdom) and used as control. After 3 days, cells were incubated with CellTiter 96 AQ_ueous_ One Solution reagent (Promega, Southampton, United Kingdom) in the dark at 37°C for up to 4 h. The reaction was stopped with 10% SDS and the absorbance was measured at 490 nm using a Hidex Sense microplate reader (LabLogic Systems Ltd., Sheffield, United Kingdom). Background absorbance was measured in wells with medium only.

### Animals and Tumors

Animal experiments were performed in accordance with the UK Home Office Animals (Scientific Procedures) Act 1986 and approved by the local ethical review panel. Male C57BL/6J mice, 6–8 weeks old (Charles River, Harlow, United Kingdom), were injected subcutaneously with 5 × 10^6^ TRAMP-C2 cells in 100 µl PBS into the right flank. Calipers were used to measure the tumor length (L), width (W) and depth (D) and the volume was calculated using the ellipsoid shape formula: (π/6) × L × W × D. When tumors reached approximately 100 mm^3^ in volume, mice were randomly divided into six treatment cohorts with *n* = 7 mice in each cohort. Mice where treated intratumorally with either 2.5 μg/dose cyto- IL-15, 5 μg/dose cyto- IL-15, 10 μg/dose cyto- IL-15, 10 μg/dose IL-15 Gen (this is the same as mod IL-15 Gen termed IL-15 Gen in the *in vivo* experiments for simplicity), 10 μg/dose cyto-IL-15 Gen or with vehicle (PBS). All IL-15 concentrations were based on protein measurements by ELISA. Treatments were given as two doses injected at days 0 and 3. Mice were monitored for weight loss, hunched posture, discomfort, and development of rashes. The survival endpoint (experimental endpoint) was when tumors reached a maximum diameter of 15 mm.

### Statistical Analysis

Data were analysed using GraphPad Prism 8 (GraphPad Software, La Jolla, CA). Statistical significance of differences was determined by one- or two-way ANOVA with Sidak’s or Dunnett’s multiple comparisons post-tests, with a 5% level of significance. Results are presented as mean ± 1 standard error of the mean (SEM).

## Results

### Production and Purification of Modified IL-15

**FIGURE 3:**
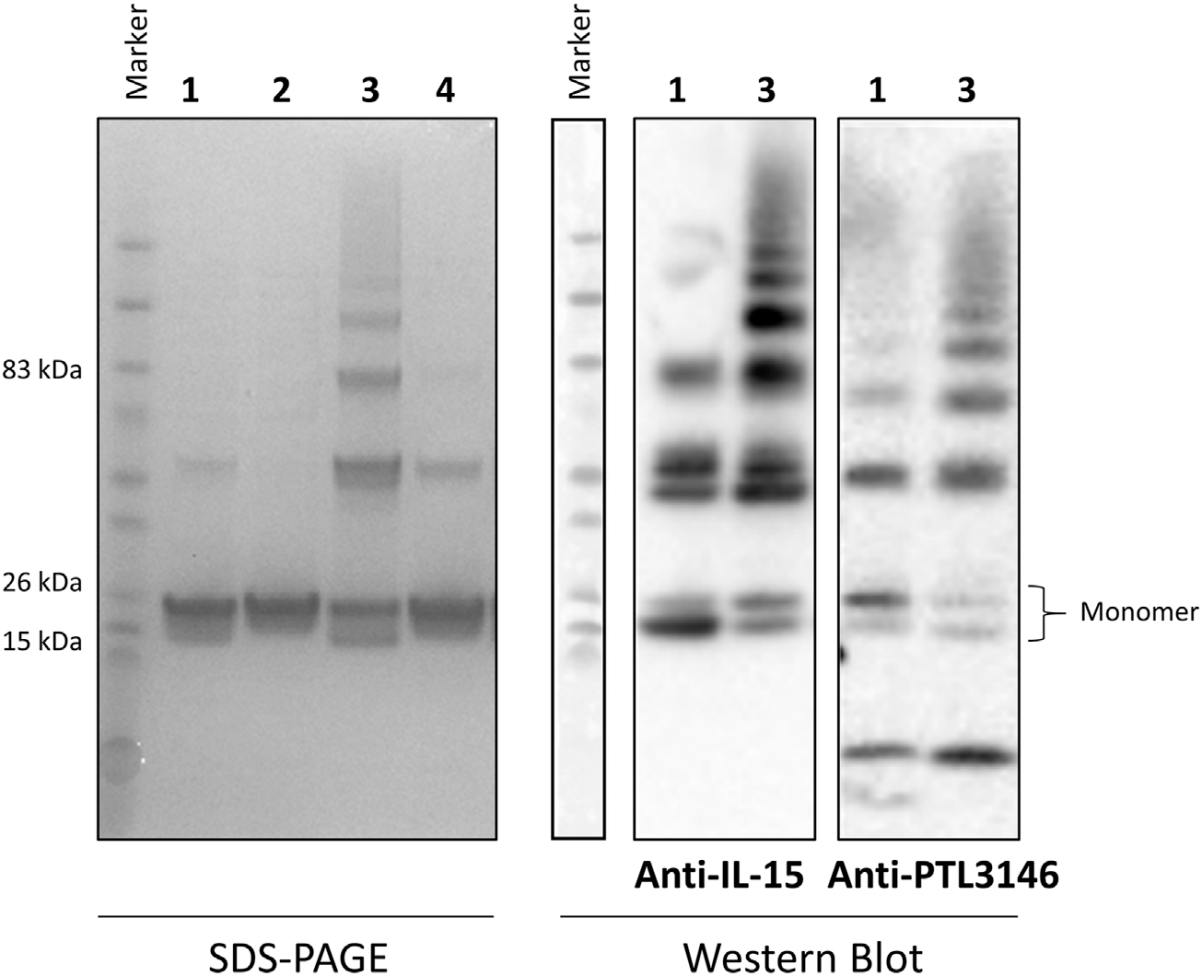
Evaluation of purity and cytotopic modification of IL-15. An SDS-PAGE gel (left) was run to evaluate the purity of the recombinant modified protein. Also, immunoblotting (right) using antibodies against IL-15 (anti-IL-15) and the peptide chain of the cytotopic molecule PTL3146 (anti-PTL3146) was performed to evaluate the efficacy of the cytotopic modification. Two batches of recombinant protein were used: protein obtained using the optimized protocol described in this paper (mod IL-15, 1) and protein obtained from inclusion bodies (mod IL-15 Gen, 3). Samples (2) and (4) correspond to the reduced forms of (1) and (3), respectively. Reduction was achieved by adding 100 mM DTT to the samples 2 and 4 before loading into the SDS-PAGE.

In previous experiments, recombinant IL-15 was obtained from inclusion bodies, resulting in low yields of protein recovered after refolding and most of the purified protein being in the form of complexes. In this study, we aimed to optimize the production and purification of a modified human IL-15 for further conjugation with a cytotopic peptide that allows IL-15 to localize at the surface of cells. Production was carried out in *E. coli* cells and the nucleotide sequence encoding mature IL-15 was optimized for expression in bacteria. Induction of expression was done at 16°C to slow down translation of the protein and, consequently, improve protein folding. However, in this study, decreasing the temperature did not lead to the production of soluble IL-15 since, after disruption of *E. coli*, no IL-15 was present in the soluble fraction (data not shown). Therefore, the supernatant, where all soluble and membrane proteins were present, was discarded and purification of IL-15 proceeded using cell debris. Before solubilisation of inclusion bodies with complete denaturing conditions (7M guanidine hydrochloride), cell debris were washed with a buffer containing 2M urea, another chaotropic agent typically used to solubilize inclusion bodies at higher concentrations (4M or higher). After three washes, pellet was treated with 7M guanidine hydrochloride in buffer and solubilization of inclusion bodies was proceed overnight. Both fractions (washing fraction and solubilized inclusion bodies) were used to purify the modified IL-15. After purification and refolding, a large amount of IL-15 was present in the wash fraction (Supplementary Figure 1 and Figure 3). From 12 g of *E. coli* biomass (1.6 L growth), we were able to purify from the washed fraction 3.4 mg of bioactive modified IL-15. Amount and/or percentage of purified IL-15 at each step of the purification for both solubilization methods are shown in Table 1. Protein concentration was estimated by BCA protein assay and ELISA assay and results were identical; however, IL-15 obtained from inclusion bodies had up to seven times lower concentration when protein was estimated by ELISA compared with that estimated by BCA protein assay. Protein estimation by BCA protein assay and ELISA is essential in this work. While BCA protein assay detects all isoforms of IL-15, with ELISA assay we were only able to measure active IL-15. This observation was validated by proliferation assays. Also, the results have shown that IL-15 purified using mild denaturing conditions was mainly present in a monomer (Figure 3, condition 1) while protein purified from inclusion bodies was forming homodimers and higher complexes (Figure 3, condition 3). When samples were incubated with a reducing agent prior to loading into an SDS-PAGE, a single band with the correct molecular weight at approximately 18 kDa was observed for both fractions (Figure 3, conditions 2 and 4). The two batches of the purified protein were used for further conjugation with the cytotopic peptide PTL3146. The binding was achieved *via* the formation of a disulfide bridge between the modified IL-15 and the cytotopic molecules. The success of this reaction was confirmed by western blot using an antibody that recognizes the peptide that is present in the cytotopic molecule (Figure 3).

**TABLE 1:** Amount of total protein and percentage of purified IL-15 at each step of the purification for both solubilization methods. Percentages were estimated from SDS-PAGE analysis.

	Total protein / IL-15 (%)			
	2M Urea		7M Gu-HCL	
	Total protein	IL-15 (%)	Total protein	IL-15 (%)
*E. coli* biomass	12 g	<1%	12 g	<1%
After cell disruption	∼6 g	n.d	∼6 g	n.d
After solubilization	n.d	47%	n.d	n.d
After HisTrap purification	3.4 mg	97%	1.1 mg	98%

a n.d. not determined.

### Characterization of Cytotopically Modified Proteins

**FIGURE 4:**
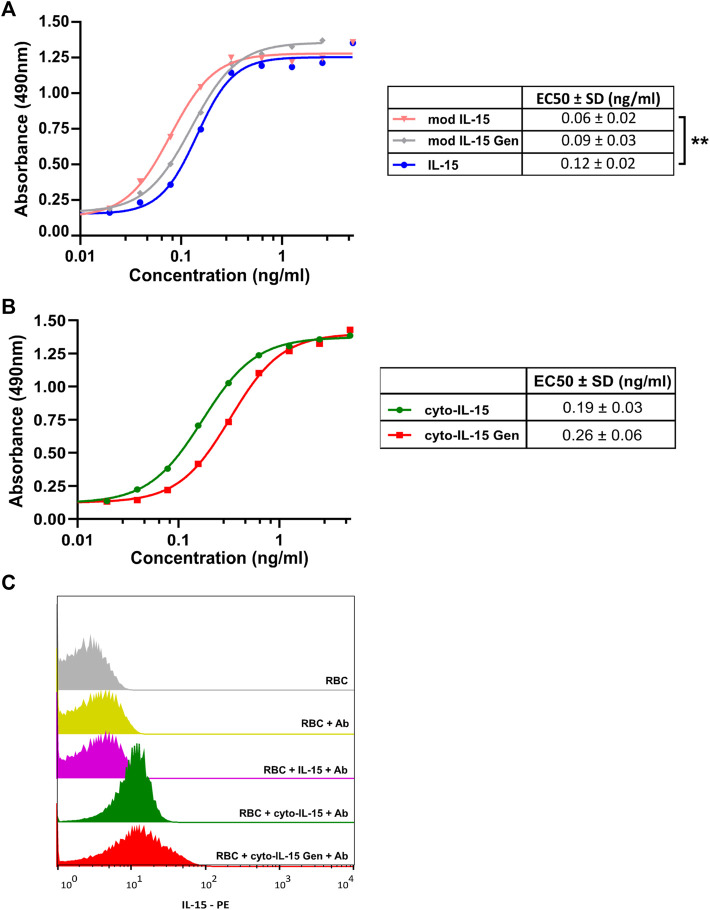
Cell proliferation and binding assays for cytotopically modified IL-15. **(A,B)** Representative cell proliferation assay carried out by incubation of different concentrations of the protein of interest with murine CTLL-2 cells for 72 h. Proliferation was quantified by measuring optical densities at 490 nm. The tables show the mean EC50s (±1 SD) for each protein calculated from individual curves from 3 to 4 independent experiments (**p < 0.01 unpaired *t*-test). **(C)** Murine RBCs were incubated with 1 μg of the protein of interest followed by incubation with an anti-IL-15 antibody conjugated with PE. Fluorescence intensity was measured using a FACSCalibur flow cytometer. Mod IL-15: modified IL-15, mod IL-15 Gen: modified IL-15 purified from inclusion bodies; Cyto-IL15, cytotopically modified IL-15; cyto-IL15 Gen, cytotopically modified IL-15 purified from inclusion bodies; IL-15, commercially available IL-15 from Peprotech.

When IL-15 binds to its receptor at the cell surface, it induces proliferation and activation of cytotoxic T lymphocytes in humans. Murine CTLL-2 cells also express the receptors for IL-15, hence, to investigate if the cytotopically modified IL-15 was active, different concentrations of the protein of interest and respective controls were incubated with CTLL-2 cells and their proliferation was measured after 3 days by optical density at 490 nm. The higher the absorbance, the higher the number of cells in the sample. Figure 4 shows results obtained before (A) and after (B) cytotopic modification. As a positive control, we used commercially available human IL-15 (IL-15, blue) (PeproTech EC Ltd). We tested the protein purified from the wash fraction before (mod IL-15, pink) and after cytotopic modification (cyto-IL-15, green), and IL-15 purified from inclusion bodies before (mod IL-15 Gen, grey) and after cytotopic modification (cyto-IL15 Gen, red). Representative curves from the assay and EC50s for each protein examined are shown. The EC50 of mod IL-15 was significantly lower than that of commercially available IL-15 (*p* < 0.01), indicating that mod IL-15 is more potent at proliferating immune cells than commercially available IL-15. The EC50 of mod IL-15 Gen was slightly higher than that of mod IL-15, but this did not reach statistical significance. When the two cytotopically modified proteins were compared, the EC50 of cyto-IL-15 appeared to be slightly lower than that of cyto-IL-15 Gen, however these were not statistically significant. Due to potential differences in antibody recognition of the cytotopically modified IL-15 versus its non-conjugated version in the ELISA used here, and differences on the molecular weight of the proteins (weight increases after cytotopic modification), the EC50s of the non-conjugated proteins were not compared with those of the cytotopically modified proteins.

The ability of the cytotopically modified IL-15 to bind to cell membranes *via* the myristoylated peptide and not *via* a receptor was also examined. Murine RBCs which do not express IL-15 receptors were used. When RBCs were incubated with purified IL-15 (mod IL-15), very low binding was measured (6.84% positive cells) (Figure 4C). This was comparable to the negative control, where no protein was added to RBCs (5.49% positive cells). On the contrary, incubation of the cells with cytotopically modified IL-15 led to an increase in binding; 70.9% positive cells with cyto-IL-15 and 64.1% positive cells with cyto-IL-15 Gen. This shows that IL-15 binds to the surface of RBC *via* the myristoylated peptide attached to IL-15.

### *In vivo* Efficacy of the Cytotopically Modified IL-15 in a Subcutaneous Prostate Tumor Model

To test the efficacy and identify the optimal dose of cyto-IL-15, mice with TRAMP-C2 prostate subcutaneous tumors were injected intratumorally with different doses of the cytotopically modified IL-15. Purified IL-15 from inclusion bodies (IL-15 Gen) and purified IL-15 from inclusion bodies after cytotopic modification (cyto-IL-15 Gen) were also used at the higher dose to compare with cyto-IL-15. Cyto-IL-15 Gen was previously shown to delay TRAMP-C2 tumor growth and increase mice survival (Papaevangelou et al., 2020).

**FIGURE 5:**
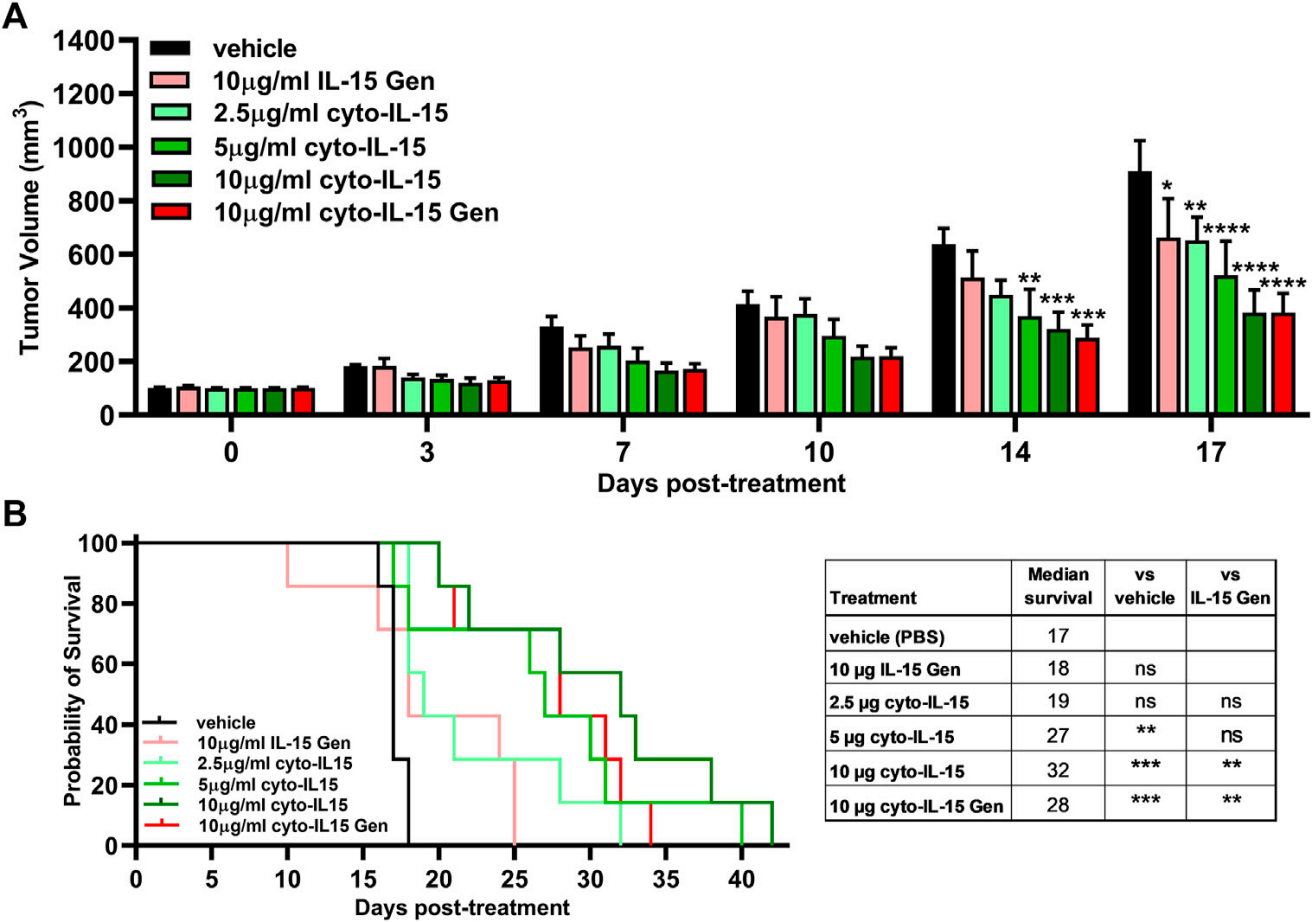
Effects of cytotopically modified *in house*–produced IL-15 on growth of TRAMP-C2 subcutaneous prostate tumors. **(A)** Tumor volumes up to day 17 post-treatment. Data are means  + 1 SEM for all the tumors per group (*p < 0.05, **p < 0.01, ***p < 0.001, ****p < 0.0001 two-way ANOVA with Dunnett’s multiple comparisons post-test). **(B)** Survival curves of mice post-treatment. The table shows the median survival of each group and comparisons of equality of individual survival curves with the survival curve of vehicle or IL-15 Gen [**p < 0.01, ***p < 0.001, Log-rank (Mantel-Cox) test]. Note: IL-15 Gen is same as mod IL-15 Gen.

By day 14 post-treatment, both the 5 and 10 µg doses of cyto-IL-15 treatments significantly delayed tumor growth in comparison with vehicle by 42% (*p* < 0.01) and 50% (*p* < 0.001) respectively. Additionally, treatment with 10 µg cyto-IL-15 Gen delayed tumor growth by 55% (*p* < 0.001) compared with vehicle. At day 17, all treatments had a significant effect on the growth of TRAMP-C2 tumors. In comparison with vehicle, 10 µg of IL-15 Gen delayed growth by 27% (*p* < 0.05) and 2.5 µg cyto-IL-15 by 28% (*p* < 0.01). However, the efficacy of cyto-IL-15 was substantially increased with doses higher than 5 µg. Five micrograms cyto-IL-15 and 10 µg cyto-IL-15 led to a growth delay of 43% (*p* < 0.0001) and 58% (*p* < 0.0001), respectively, compared with vehicle. A significant delay in growth was also seen with 10 µg cyto-IL-15 Gen by 58% (*p* < 0.0001) compared with vehicle, with the effect being the same as the one seen with the same dose of cyto-IL-15 (Figure 5A and Supplementary Figure 2A).

Despite the small effect on tumor growth caused by IL-15 Gen or low dose (2.5 µg) of cyto-IL-15, only cyto-IL-15 in doses higher/equal to 5 µg had an effect on mice survival. Five micrograms cyto-IL-15, 10 µg cyto-IL-15 and 10 µg cyto-IL-15 Gen significantly increased median survival to 27 (*p* < 0.01), 32 (*p* < 0.001) and 28 days (*p* < 0.001) respectively, compared with vehicle (17 days). Survival of mice treated with 10 µg cyto-IL-15 or cyto-IL-15 Gen was also significantly longer compared with IL-15 Gen (*p* < 0.001) (Figure 5B). Intratumorally injected IL-15, cytotopically modified or not, was well tolerated, and none of the mice suffered side effects, such as rashes or weight loss (Supplementary Figure 2B). The therapeutic potential of cyto-IL-15 versus cyto-IL-15 Gen is summarized in Supplementary Table 1.

## Discussion

In the present study, we have demonstrated an alternative way to produce and purify IL-15, where a washing step with 2M urea was used to recover the largest amount of IL-15 while a smaller amount was obtained after solubilization of inclusion bodies with 7M guanidine hydrochloride. This new approach resulted in a high yield of purified IL-15 (2.1 mg/L from washing step vs. 0.7 mg/L from inclusion bodies). High concentration of the purified protein is important when performing *in vivo* experiments with intratumoral agent delivery, as only a small volume can be injected in the tumor (approximately 50 µl), and circumvents the need for a further protein concentration step that may lead to additional protein aggregation and losses. The purified IL-15 was then conjugated to a cytotopic peptide to allow anchoring of IL-15 to cell membranes (cyto-IL-15) and its activity and efficacy were investigated *in vitro* and *in vivo* in a prostate cancer murine model. Our key findings indicate that functional IL-15 can be inexpensively produced and purified yielding large amounts of protein by using a urea wash step. Moreover, cytotopic modification of the purified IL-15 results in a highly active protein that can significantly prolong mouse survival by delaying tumor growth without any side effects. This is in agreement with what has been reported previously (Papaevangelou et al., 2020). A similar study to optimize the production of recombinant IL-15 in *E. coli* cells was performed by Ward and co-authors (Ward et al., 2009). From 1 L of TB culture, the authors were able to purify approximately 1.3 mg of active IL-15 (Supplementary Table 2). A second study performed by Vyas et al. (2012) was carried out. The authors to produce hIL-15 in large scale for clinical studies. Growth was performed in 20 L fermentation vessels containing 10 L production medium. From a 10 L growth, the authors claimed to obtain from 196 to 342 mg of IL-15, depending on the *E. coli* strain that was used.

Cytotopic modification using a myristoylated peptide enables the localization of pharmacologically relevant molecules on the surface of cells by spontaneous anchoring of the peptide in the cell membrane (Smith, 2002; Karegli et al., 2017). Cytotopic modification is the process when a cytotopic molecule is attached/conjugated to a therapeutic agent. The cytotopic molecule contains a fatty acid chain, which facilitates the interaction of the molecule with the cell membrane, providing the therapeutic agent the ability to localize on the cell surface. For instance, a membrane-localizing form of the human complement receptor type 1, Mirococept, and hirudin-like peptide, Thrombalexin 1, have been developed in the past (Patel et al., 2006; Karegli et al., 2017; Kassimatis et al., 2017). Mirococept can prolong survival of donor kidneys in rats and has been used to prevent delayed graft function in a clinical trial of renal transplantation, while thrombalexin 1, was used to inhibit thrombin and prevent acute antibody-mediated thrombosis in donor organs in a rat model of hyperacute rejection (Patel et al., 2006; Kassimatis et al., 2017). Recently, the effect of cytotopic IL-15 in the reduction of prostate cancer tumors in mice models has been demonstrated by our laboratory (Papaevangelou et al., 2020). The injection of cytotopically modified IL-15 intratumorally in prostate tumors has been proven successful in the reduction of tumor size and in prolonging mice survival. However, the IL-15 utilized in this study was obtained from inclusion bodies. The amount of IL-15 recovered from inclusion bodies was not ideal and, as observed by SDS-PAGE ran under non-reducing conditions, the protein was forming higher complexes (Figure 3, lane 3). Therefore, it was important to improve the recombinant production and purification of IL-15 for further cytotopic modification and use *in vivo* studies.

Herein, we have used *E. coli* cells to express IL-15 with additional amino acids at the C-terminus of the protein to allow cytotopic modification of the cytokine. We successfully managed to recover bioactive protein from cell debris using 2M of urea. At this concentration, urea is considered a mild denaturing agent; therefore, we hypothesize that IL-15 probably adopted a native-like structure in the aggregates, resulting in the recovery of most of the protein in a biologically active form (Palmer and Wingfield, 2004; Singhvi et al., 2020). We concluded that most of the protein was active since the total protein amount was estimated both by BCA protein assay and IL-15 ELISA and the results were identical. The protein concentrations estimated by ELISA were used to observe the proliferation of CTLL-2 cells, and the EC50 for purified mod IL-15 was found to be lower than that of the positive control (human IL-15 from Peprotech). Purification of bioactive proteins from inclusion bodies using mild-denaturing conditions have been reported in the literature. Human granulocyte-colony stimulating factor (hG-CSF) was extracted from inclusion bodies with the correct folding using mild-denaturing conditions (Jevsevar et al., 2005; Singh et al., 2015). In the past, human IL-15 produced in *E. coli* was recovered from inclusion bodies (Ward et al., 2009; Vyas et al., 2012), which hinders the process of recovering biologically active protein and reduces the yield of protein. In *E. coli*, the cytoplasm has a reducing environment, inhibiting the formation of disulfide bonds in proteins (Stewart et al., 1998). Since the mature polypeptide chain of IL-15 contains four cysteine residues, forming two disulfide bonds between Cys35 and Cys85 and Cys42 and Cys88 (Chirifu et al., 2007), this may be one of the reasons why the protein tends to aggregate and form inclusion bodies when expressed in *E. coli* (Gaciarz et al., 2017). In the current study, by using urea at low concentrations, we managed to produce bioactive IL-15 that was subsequently cytotopically modified to be used in further *in vivo* studies.

The *in vivo* investigation of the efficacy of IL-15 showed that only cytotopically modified IL-15 in doses equal or higher to 5 µg led to both a significant growth delay of TRAMP-C2 prostate tumors and an increase in mice survival compared with control. This effect was greater than treatment with IL-15 Gen (no cytotopic modification) or low dose of cyto-IL-15 (2.5 µg), as these led to a small delay in tumor growth at day 17 compared with control but did not increase mice survival. Interestingly, the higher dose of cyto-IL-15 (10 µg) derived from IL-15 purified using mild denaturing conditions had a similar effect in terms of tumor growth delay with the same dose of cyto-IL-15 Gen derived from IL-15 recovered from inclusion bodies (cyto-IL-15 Gen) and a slightly greater effect in terms of mice survival (32 vs. 28 days).

In conclusion, a modified version of IL-15 was inexpensively and efficiently expressed in *E. coli* and purified using mild denaturing conditions. After the cytotopic modification the protein remains active, and the *in vivo* results have shown that the efficacy of the protein in the treatment of prostate tumors in mice is comparable to what has been previously reported by our laboratory (Papaevangelou et al., 2020). The use of mild denaturing conditions allowed us to improve our ability to produce and purify IL-15, a non-trivial protein. Moreover, this study further highlights the potential of IL-15 as an effective immunotherapy for the treatment of patients with prostate cancer.

## Data Availability

The original contributions presented in the study are included in the article/Supplementary Material, further inquiries can be directed to the corresponding authors.

## Abbreviations

DTT, dithiothreitol; FBS, fetal bovine serum; HRP, horseradish peroxidase; hG-CSF, human granulocyte-colony stimulating factor; IL-15, interleukin-15; IPTG, isopropyl β-d-1-thiogalactopyranoside; NK cells, natural killer cells; OD, optical density (OD); PBS, phosphate buffered saline; RBC, sred blood cells; RT, room temperature; SEM, standard error of the mean; TCEP, tris(2-carboxyethyl) phosphine; TRAMP, transgenic adenocarcinoma of the mouse prostate; γ_c_, common gamma chain.
