# Supplementary material for: Cytotopic (Cyto-) IL-15 as a New Immunotherapy for Prostate Cancer: Recombinant Production in Escherichia coli and Purification
Source: Front Mol Biosci. 2021 Oct 27;8:755764. doi: 10.3389/fmolb.2021.755764 (PMC8578882; doi:10.3389/fmolb.2021.755764)
Supplement: Supplementary file 1 [file Presentation1.pdf]

Supplementary Materials

Supplementary Figures

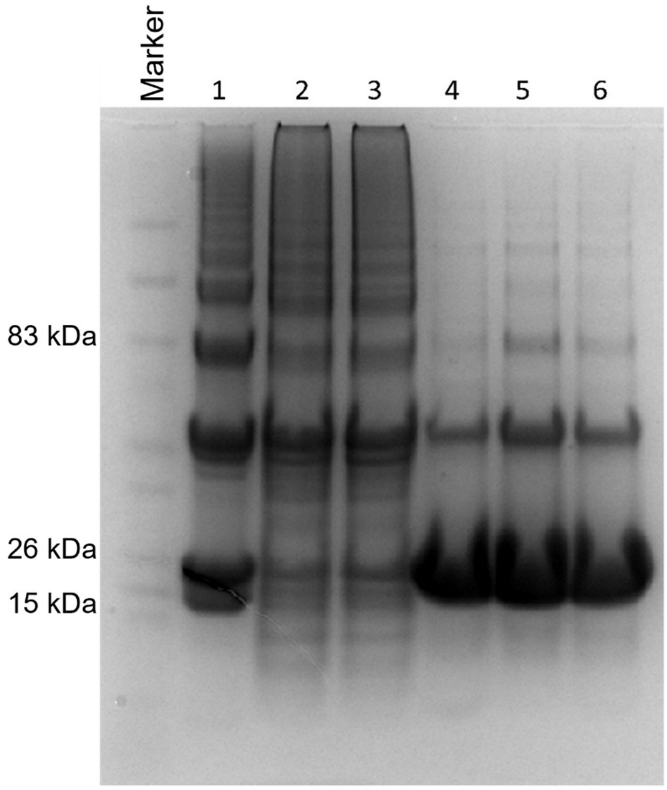

Supplementary Figure 1. Purification of solubilized IL-15. Lane 1, Batch of mod IL-15 Gen (produced by Genscript); Lane 2 and 3, fractions from Histrap after loading protein solubilized overnight with 7M guanidine hydrochloride (protocol provided by Genscript); Lane 4 to 6, fractions from Histrap after loading protein solubilized with 2M urea (washing step) (mod IL-15).

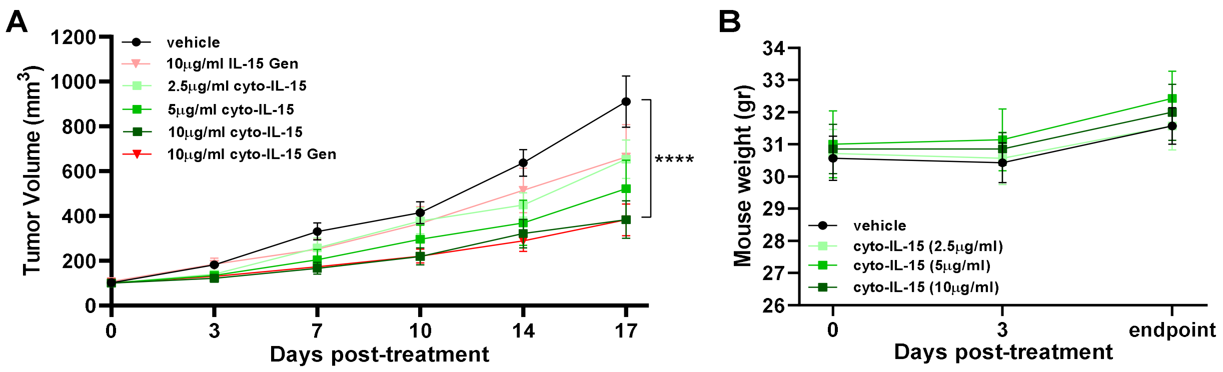

Supplementary Figure 2. Effects of cytotopically modified *in house*–produced IL-15 on TRAMP-C2 tumor growth and mice weight. (A) Tumor growth curves up to day 17 post-treatment. Data are means ± 1 SEM for all the tumors per group (****p <0.0001 two-way ANOVA with Dunnett's multiple comparisons post-test). (B) Mice weights of all mice per group until survival endpoint. Note: IL-15 Gen is same as mod IL-15 Gen.

Supplementary Tables

Supplementary Table 1. Comparison of therapeutic potential between cyto-IL-15 (modified IL-15 obtained from the washings with mild denaturing buffer) and cyto-IL-15 Gen (modified IL-15 obtained from solubilization of inclusion bodies).

	**Cyto-IL-15**	**Cyto-IL-15 Gen**
**Ratio of protein concentration measured by ELISA versus BCA protein assay**	1	0.14
**EC50 for CTLL-2 cells (ng/ml)**	0.19 ± 0.03	0.26 ± 0.06
**Tumor growth delay at day 17 post-treatment (%) ***	58	58
**Median mouse survival post-treatment (days) ***	32	28

*All mice were treated with equivalent doses (10mg) of either cyto-IL-15 or cyto-IL-15 Gen, based on ELISA concentrations.

Supplementary Table 2. Production of recombinant human IL-15 reported in the literature.

**Author**	**Host**	**Medium/ Growth**	**Culture conditions**	**Localization**	**Protein yield**
Ward et al. 2009	E. coli BL21 (DE3) star	TB.<br><br>Growth in flasks.	Induction with 0.4 mM IPTG at OD (600 nm) 0.6. Growth continued overnight at 37°C.	Inclusion Bodies	1.32 mg/L
Vyas et al. 2012	BL21-AI	TB medium with small modifications.<br><br>Growth in 20L fermentation vessel.	When cell density reached 4–10 (OD 600nm), IPTG and arabinose were added to induce expression. Three hours after the induction, cells were harvested by centrifugation.	Inclusion Bodies	19.6 mg/L
Our study	*E. coli* BL21 star (DE3)	TB.<br><br>Growth in flasks.	Induction with 0.5 mM IPTG when OD at 600 nm reached 0.6. Growth overnight at 16°C.	Native-like Inclusion Bodies	2.1 mg/L
